# The Effects of Realistic Synaptic Distribution and 3D Geometry on Signal Integration and Extracellular Field Generation of Hippocampal Pyramidal Cells and Inhibitory Neurons

**DOI:** 10.3389/fncir.2016.00088

**Published:** 2016-11-08

**Authors:** Attila I. Gulyás, Tamás F. Freund, Szabolcs Káli

**Affiliations:** Laboratory of Cerebral Cortex Research, Institute of Experimental Medicine, Hungarian Academy of SciencesBudapest, Hungary

**Keywords:** intracellular recording, field potential, synaptic inputs, synaptic currents, multicompartmental modeling, perisomatic inhibition

## Abstract

*In vivo* and *in vitro* multichannel field and somatic intracellular recordings are frequently used to study mechanisms of network pattern generation. When interpreting these data, neurons are often implicitly considered as electrotonically compact cylinders with a homogeneous distribution of excitatory and inhibitory inputs. However, the actual distributions of dendritic length, diameter, and the densities of excitatory and inhibitory input are non-uniform and cell type-specific. We first review quantitative data on the dendritic structure and synaptic input and output distribution of pyramidal cells (PCs) and interneurons in the hippocampal CA1 area. Second, using multicompartmental passive models of four different types of neurons, we quantitatively explore the effect of differences in dendritic structure and synaptic distribution on the errors and biases of voltage clamp measurements of inhibitory and excitatory postsynaptic currents. Finally, using the 3-dimensional distribution of dendrites and synaptic inputs we calculate how different inhibitory and excitatory inputs contribute to the generation of local field potential in the hippocampus. We analyze these effects at different realistic background activity levels as synaptic bombardment influences neuronal conductance and thus the propagation of signals in the dendritic tree. We conclude that, since dendrites are electrotonically long and entangled in 3D, somatic intracellular and field potential recordings miss the majority of dendritic events in some cell types, and thus overemphasize the importance of perisomatic inhibitory inputs and belittle the importance of complex dendritic processing. Modeling results also suggest that PCs and inhibitory neurons probably use different input integration strategies. In PCs, second- and higher-order thin dendrites are relatively well-isolated from each other, which may support branch-specific local processing as suggested by studies of active dendritic integration. In the electrotonically compact parvalbumin- and cholecystokinincontaining interneurons, synaptic events are visible in the whole dendritic arbor, and thus the entire dendritic tree may form a single integrative element. Calretinin-containing interneurons were found to be electrotonically extended, which suggests the possibility of complex dendritic processing in this cell type. Our results also highlight the need for the integration of methods that allow the measurement of dendritic processes into studies of synaptic interactions and dynamics in neural networks.

## Introduction

Our image of neurons and their integrative processes grows ever more complex thanks to significant improvements in technology. Dendritic patch-clamp and multiphoton imaging and manipulation techniques have revealed the complexity and importance of dendritic processing in cortical pyramidal neurons. Much less is currently known about dendritic integrative processes in interneurons, although some results do suggest that their dendrites may also have non-linear properties and may have roles beyond the faithful transmission of synaptically evoked signals to the axosomatic output region (Saraga et al., 2003; Chiovini et al., 2014). Meanwhile, a set of studies examined the interactions of specific cell types in cortical networks, with the aim of understanding the generation of different kinds of population dynamics such as coherent oscillations and transient synchrony (Butler and Paulsen, 2015; Gulyás and Freund, 2015). These studies employed somatic patch-clamp recordings and extracellular electrode arrays *in vivo* and *in vitro* (Ylinen et al., 1995; Lakatos et al., 2005; Mann et al., 2005; Oren et al., 2006, 2010; Montgomery et al., 2009; Makarov et al., 2010; Sullivan et al., 2011; Scheffer-Teixeira et al., 2012, 2013), which allow recording from all layers of a structure and the calculation of currents flowing in and out of neurons during different activity patterns. Several recent papers, using complex recording methods and data analysis, dissected how the activity of different identified cell types (Mann et al., 2005; Oren et al., 2006, 2010; Hájos et al., 2013) and input pathways (Isomura et al., 2006; Montgomery et al., 2009) contribute to the generation of network activity, and how excitatory and inhibitory synaptic currents and voltage-gated currents shape neuronal activity and field potentials (Buzsáki et al., 2012).

Although both somatic voltage clamp (VC) recordings and extracellular field potential measurements provide some information about synaptic inputs, the relationship between these various measures and the actual synaptic current is not in fact straightforward due to the attenuation and complex interactions of these signals within and across neurons. The question of how accurately these experiments can measure synaptic inputs, and how this depends on the characteristics of the cells such as their morphology or the locations of the inputs, has not been examined systematically. These factors would only be negligible if neurons were well-approximated by an electrotonically compact cylinder with uniform distributions of excitatory and inhibitory inputs (Trevelyan and Watkinson, 2005; Trevelyan, 2009). This model is incorrect at three points: (1) neurons are not electrotonically compact; (2) synaptic inputs are not evenly distributed over the surface of cells, and the distribution is cell type specific; (3) neuronal processes intermingle in 3D, and thus cells may cancel their own extracellular signal and the signal of other cells.

Williams and Mitchell (2008) made an exhaustive attempt to examine the first point. In a heroic study involving double and triple patch-clamp recordings from single layer 5 pyramidal cells (PCs) in the VC and current clamp configurations, they measured how space clamp efficiency and current recovery in VC break down away from the soma. Due to technical limitations they could not go further than 600 μm out on the main apical dendrite of the cells. The sobering result was that even for inputs to these rather thick, and therefore electrotonically compact apical dendrites only 20% of the injected current was recovered at the soma. Marchionni and Maccaferri (2009), when trying to estimate the strength of dendritic versus somatic inhibition during epileptiform activity, also demonstrated how quickly VC breaks down away from the soma and misses the currents to be measured. These results suggest that the measurement error of synaptic currents in second order thin dendrites, which constitute the major part of the PC dendritic arborization, should be very large.

In order to determine the relationship between actual synaptic inputs and their various intra- and extra-cellular measures, estimate the extent of observation errors and study the differences in the integration strategy of cell types, here we use multicompartment neuronal models of different cell types in area CA1 of the rat hippocampus. We model synaptic currents in CA1 PCs and three distinct inhibitory neuron (IN) types [parvalbumin- (PV), cholecystokinin- (CCK), and calretinin- (CR) positive inhibitory cells] using realistic dendritic geometry and synapse distributions to quantify errors in space clamp and current recovery. We also examine using 3D models of PCs the consequences of the observed excitatory and inhibitory input distributions on the generation of local field potentials (LFPs).

In the behaving animal brain states associated with different behaviors are accompanied by distinct EEG patterns, which indicate different neuronal dynamics characterized by unique, fluctuating levels of activity and synchrony (Buzsaki, 1989). The electrotonic properties and thus the integrative processes of a neuron are strongly sensitive to the conductance of the membrane. During high activity or synchronous states neurons are bombarded with strong excitatory and inhibitory inputs. These phasic and tonic currents decrease the input resistance of the neuron significantly and change its integrative properties. In order to understand how brain states influence signal propagation and input integration in neurons, we examine the above questions in the absence of background synaptic input as well as assuming different realistic activity levels.

We argue that such a quantitative assessment of the origins of the measured signals is necessary for the correct interpretation of experimental results. For instance, a frequent conclusion based on somatic intracellular measurements is that the correlation of PC activity with network dynamics is driven primarily by inhibition, while IN activity is driven more by excitation (Oren et al., 2006, 2010; Adesnik and Scanziani, 2010; Tahvildari et al., 2012; Hájos et al., 2013). Another recurring conclusion is that perisomatic inhibition is the key player in shaping field potentials and rhythmic network activity. Our results suggest that the interpretation of some of these experiments may be biased by biophysical effects that emphasize the contributions of the perisomatic region, and the role of dendritic processes (in both the anatomical and the physiological sense) might be strongly under-estimated.

## Materials and Methods

### Neuronal Morphologies and Synapse Distributions

We selected morphological reconstructions of four types of neuron from rat hippocampal area CA1. These morphologies have been described in detail (Gulyás et al., 1999; Megías et al., 2001; Mátyás et al., 2004), and are available from the online database NeuroMorpho.Org under “Hippocampal cells from Gulyas.” We used morphology pc1a for the PC, pv08d for the PV-positive basket cell, cck7635 for the CCK-containing cell, and cr10b for the CR-positive interneuron. Briefly, the workflow of reconstruction (as detailed in the papers) was as follows. First, the dendritic morphology of selected neurons was recorded in 3D. Instead of recording the highly variable dendritic diameter for each location, we divided the dendritic arbor within each layer into thin, medium and thick segments. The same types of segments of different cells were then serially sectioned from material optimized for electron microscopical staining. GABAergic inputs were identified using post-embedding immunostaining. The excitatory and inhibitory input density were measured for each type of dendrite from long serial sections. The light microscopical dendritic length data were then multiplied by the density values to get the amount of total input and the ratio of excitation to inhibition. As result of the sampling strategy the final density values do not change continuously over the surface of the neurons, but there are discrete steps in these values and in the *E*/*I* ratios as visible in **Figure 3**.

The density of excitatory and inhibitory synapses per unit length of dendrite (or unit surface of soma) was also described in the papers above for each layer and type of dendrite, and we used these values to define the density of synapses per unit membrane area in each section of the reconstructed neurons.

### Compartmental Modeling

Multicompartmental models were created by reading the reconstructed morphologies into the Neuron simulation tool (Carnevale and Hines, 2006), and adding appropriate, spatially uniform membrane and intracellular properties (specific capacitance, membrane conductance, and axial resistance) to match the basic subthreshold physiological properties (somatic input resistance and membrane time constant) of each cell type based on the online database Hippocampome.org (Wheeler et al., 2015). The resulting values of the passive parameters were as follows: *C*_m_ = 0.01 F/m^2^, *g*_l_ = 0.8 S/m^2^, *R*_a_ = 1 Ωm for the PC; *C*_m_ = 0.01 F/m^2^, *g*_l_ = 0.5 S/m^2^, *R*_a_ = 1 Ωm for the PV-positive cell; *C*_m_ = 0.005 F/m^2^, *g*_l_ = 0.1 S/m^2^, *R*_a_ = 2 Ωm for the CCK-positive cell; *C*_m_ = 0.01 F/m2, *g*_l_ = 0.5 S/m^2^, *R*_a_ = 1 Ωm for the CR-positive cell. The altered values for *C*_m_ and *R*_a_ in the CCK cell compared to the other cell types were required to fit the reported values of input resistance and time constant in this cell type. Different values for the leak conductance were used in simulations of non-silent (low- and high-activity) states to account for the tonic effects of synaptic conductances, as described below.

### Morpho-Electrotonic Transform

The morpho-electrotonic transform (Zador et al., 1995) is a graphical representation of the electrotonic structure of the cell. In this approach, the reconstructed morphology of the cell is distorted such that it expresses functional properties such as the attenuation (or delay) of signals in the dendritic tree. The topology of the tree and the orientation of individual dendritic segments are preserved, while the anatomical length of each segment is replaced by a length which is proportional to one of these functional measures. In the example shown in **Figure 3D**, anatomical length was replaced by a distance proportional to the logarithm of voltage attenuation toward the soma at a frequency of 100 Hz, where the unit corresponds to the distance over which the amplitude of a 100 Hz sinusoidal signal decays e-fold.

### Calculation of Synaptic Conductances in Silent, Low Activity, and High Activity States

In the silent case, we activated only a single synapse with a bi-exponential time course (rise time: 0.3 ms, decay time: 3 ms) either with a non-physiologically low maximal conductance (10 pS) or with a physiologically relevant conductance (1 nS) (Magee and Cook, 2000; Bartos et al., 2002; Kubota et al., 2015). The reversal potential was assumed to be 0 mV for excitatory and –70 mV for inhibitory synapses. In the silent case, the net membrane conductance in all other parts of the neuron equals the membrane leak conductance. In the physiological (active) network states membrane conductance is estimated by taking into account the average excitatory and inhibitory synaptic conductance in each part of the cell.

To estimate the number of concurrently active synaptic inputs in the low activity state, we estimated the percentage of activated excitatory, perisomatic inhibitory, and other inhibitory synapses in a 10 ms window, based on cell-type-specific firing rates measured between sharp wave-ripple (SWR) events in brain slices showing spontaneous SWR activity (Hájos et al., 2013). Specifically, we estimated the average input rate for excitatory inputs to be 0.75 Hz, while the input rate was set to 40 Hz for perisomatic and 15 Hz for dendritic inhibitory inputs. We then multiplied the activity rate of different types of inputs (deriving from subsets of different neurons, with known firing rates) with the number and distribution of different synapses over the surface of the examined neurons, and the charge carried by a single synaptic event (using realistic conductances for each input). The estimation was repeated in a similar manner for the high activity state, where synaptic activation rates were calculated from the activation frequency of different neurons observed during the peak period of SWRs (resulting in input rates of 3 Hz for excitatory, 80 Hz for perisomatic inhibitory, and 30 Hz for dendritic inhibitory inputs).

### Model Instrumentation and Simulation

Simulated voltage-clamp experiments used an idealized setup consisting of a voltage source in series with a small resistance (10 kΩ), as implemented by the SEClamp object in the Neuron simulator, connecting the extracellular space (which is assumed to be isopotential at 0 mV) to the intracellular part of a single somatic compartment. This solution allowed us to focus on VC artifacts which are intrinsic to the neuron rather than resulting from the measurement apparatus. More realistic (single- or two-electrode) VC setups would introduce further, instrumentation-specific artifacts, which are not addressed in this study.

For compartmental simulations, models were spatially discretized using the d-lambda rule of the Neuron simulator, so that all compartments had an electrotonic length which was less than 0.1 times the space constant (lambda), which was calculated in a neuron-, location-, and state-specific manner. Simulations were run in the Neuron simulator using a fixed time step of 0.25 ms.

### Calculation of Extracellular Potentials and Current Source Density

Current source density was estimated by adding the membrane currents of dendritic segments in 3D voxels of equal size, which collectively covered the neuron. The extracellular field potential was calculated at a series of equally spaced points along a line parallel to the main axis of the pyramidal neuron, at a distance of 300 μm from the soma, by adding the contributions of all membrane currents in each section of the neuron, using the standard formula for a homogeneous, isotropic extracellular space (Nunez and Srinivasan, 2005).

## Results

In order to highlight the factors that influence signal propagation, integration and the extracellular field contribution of hippocampal neurons, we first review the trends of afferent and efferent connectivity among hippocampal CA1 PCs and different types of INs. These parameters will be then incorporated into morphologically detailed compartmental model neurons to examine how the input organization and the dendritic structure influence signal propagation and the error of recovering synaptic inputs in somatic measurements. Finally, we will pursue how the termination strategy of different cell types shapes LFP generation by PCs.

### Inhibition Is Centered Perisomatically, Especially on Principal Neurons

**FIGURE 1:**
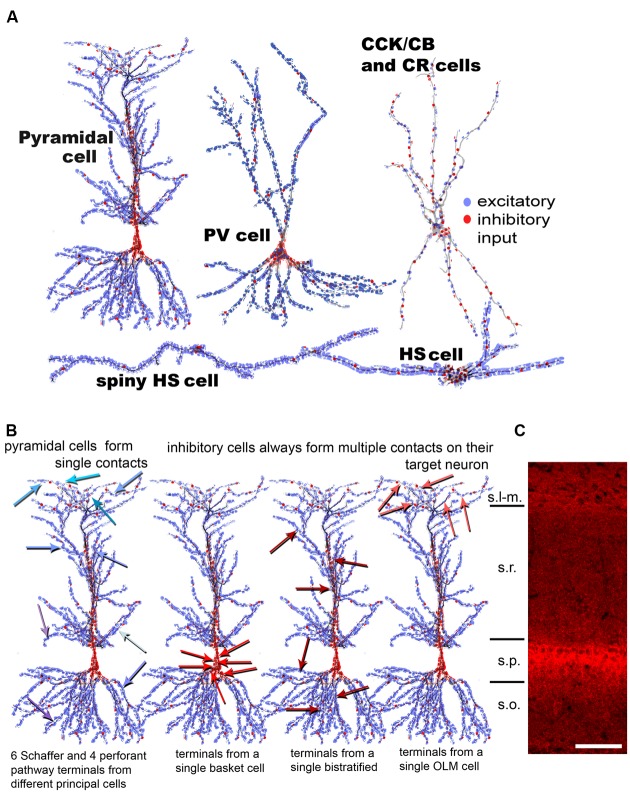
**Summary of observed synaptic distribution on different cell types of the hippocampal CA1 area. (A)** The distribution of excitatory (blue) and inhibitory (red) terminals is shown schematically over different CA1 neurons. CCK, Calbindin D28k (CB) and CR cells have a similar input organization; therefore, they are represented by a single drawing. Hippocampo-septally projecting neurons (HS cells) receive the highest amount of excitation and the lowest amount of inhibition. **(B)** The distribution of input terminals from different types of presynaptic neurons onto a CA1 pyramidal cell (PC) is shown here. Excitatory input from CA3 PCs onto CA1 PCs in str. radiatum and oriens (Schaffer collaterals) and from the entorhinal cortex in str. lacunosum-molaculare are realized via single contacts. Single contacts from many different individual excitatory axons are indicated with arrows in different shades of blue on the left drawing. In contrast, all examined inhibitory neuron (IN) populations form multiple synapses (6–10) on their target PCs, by either clustering on the soma (second cell) or scatter individually over the dendritic domains (third and fourth cells). The data presented in the figure is a summary of results from our earlier anatomical results (Gulyás et al., 1999; Megías et al., 2001; Mátyás et al., 2004; Takács et al., 2008). **(C)** Fluorescent immunostaining against the vesicular GABA transporter (courtesy of Gábor Nyíri) reveals the distribution of GABAergic terminals in the CA1 area of the hippocampus. The panel shows that the density of inhibitory terminals is highest in and close to str. pyramidale (perisomatically) and in st. lacunosum-moleculare (the termination zone of the perforant path fibers). Scale 100 μm. s.l-m.:stratum lacunosum-moleculare, s.r.: stratum radiatum, s.p.: stratum pyramidale, s.o.: stratum oriens.

**FIGURE 2:**
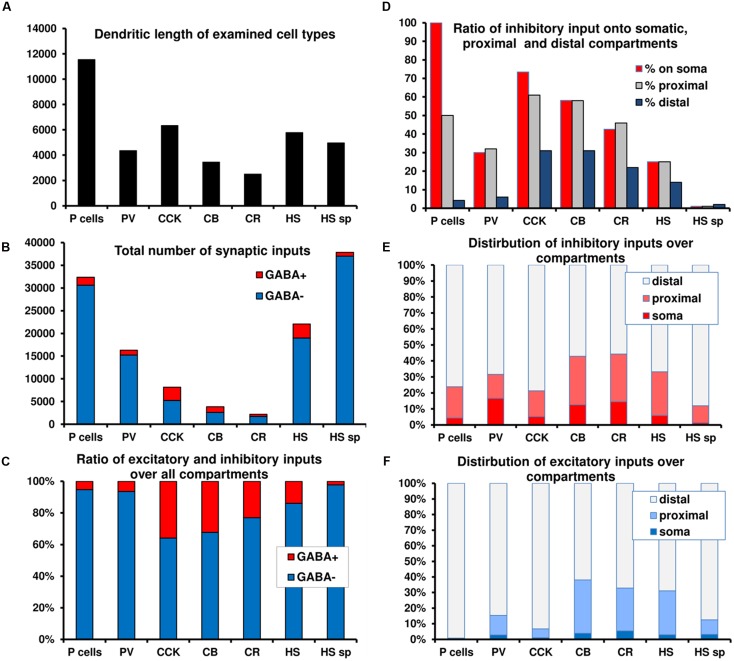
**Summary graphs of the distribution of excitatory and inhibitory inputs over different domains of CA1 neurons. (A–C)** Total length, absolute and relative amount of excitation and inhibition over seven cell types. Note the large difference in the amount of converging inputs and in the ratio of inhibitory inputs. **(D)** Ratio of inhibitory inputs over different compartments of the cell types. Note the increase in the ratio of inhibition toward the soma, which, in the extreme case of PCs means that no excitation arrives somatically. **(E–F)** The distribution of excitatory and inhibitory inputs among compartments for the different cell types. A significant portion of inhibition arrives proximally for most neurons. In the case of PCs virtually all excitation arrives distally. The data presented in the figure is a summary of results from our earlier anatomical results (Gulyás et al., 1999; Megías et al., 2001; Mátyás et al., 2004; Takács et al., 2008).

The distribution of excitatory and inhibitory synaptic inputs (identified by postembedding GABA immunostaining at the electron microscopical level) over key populations of rat hippocampal CA1 area neurons: PCs, PV-, CCK-, Calbindin D28k-, and CR-positive as well as two types of hippocampo-septally projecting (HS) INs has been quantitatively described (Gulyás et al., 1999; Megías et al., 2001; Mátyás et al., 2004; Takács et al., 2008). **Figure 1A** compares schematically the relative distribution of excitatory (blue) and inhibitory (red) inputs over the domains of different cell types in different hippocampal layers, innervated by inputs of different origin. The quantitative conclusions are summarized in the charts of **Figure 2**. **Figure 1B** summarizes the termination strategy of excitatory as well as three different types of inhibitory inputs on CA1 PCs.

The fundamental organizational similarities and differences among cell types, in the order of their importance for intracellular signal propagation and LFP generation, are as follows:

- a significant portion of inhibition arrives onto the soma and proximal dendrites in all examined neuron classes (**Figure 2E**), because the density of inhibitory inputs strongly increases toward the soma (**Figure 2D**);
- PCs receive only inhibitory and no excitatory inputs onto their somata and mostly inhibitory inputs onto their proximal dendrites (**Figures 2E** vs. **2F**);
- excitatory inputs of PCs predominantly arrive onto electrotonically remote, second-order (oblique) spiny dendrites, which receive a low percentage of inhibitory input (**Table 1**);
- INs receive both excitatory and inhibitory inputs perisomatically (**Figures 2E** vs. **2F**);
- the thick apical dendritic trunk of PCs anchors (geometrically and electronically) the much thinner second order apical dendrites to the soma (Megías et al., 2001), and can be considered as a functional (electrotonic and anatomical) extension of the soma (see below);
- the amount of synaptic input and the ratio of inhibitory synapses is highly variable among different cell types. PCs and PV neurons have similar input organization, with many excitatory inputs and comparatively weak inhibition. CCK, CR, and CB cells receive less input and have a higher inhibitory ratio (**Figures 2B,C**).
- HS cells (not modeled in the current study) receive an exceptionally low amount of inhibitory inputs, even onto their somata (Takács et al., 2008) (**Table 1**).

**Table 1:** Absolute and relative distributions of excitatory and inhibitory synaptic inputs over the surfaces of seven examined hippocampal neuron types.

	Total dendritic length (μm)	Total inputs	Excitatory inputs	Inhibitory inputs	Inhibitory ratio (%) total	somatic (%)	peri-somatic (%)	distal dendritic (%)
P cells	11500	**32400**	30600	1700	5.3	**100.0**	50.0	4.2
PV	4300	16300	15200	1100	6.0	30.0	32.0	6.0
CCK	6300	8100	5200	3000	**36.0**	73.4	61.0	**31.0**
CB	3400	3800	2600	1200	29.0	58.1	58.0	**31.0**
CR	2500	***2200***	1700	500	20.0	42.5	46.0	22.0
HS	5800	22000	19000	5300	14.0	25.0	25.0	14.0
HS sp	5000	**37700**	37000	0	***2.3***	***1.0***	1.0	***2.0***

	**Relative distribution of excitation (%)**			**Relative distribution of inhibition (%)**		
	**soma**	**proximal**	**distal**	**soma**	**proximal**	**distal**

P cells	***0.0***	0.8	**99.2**	4.3	19.6	76.1
PV	2.8	12.5	84.6	16.6	15.0	68.4
CCK	1.0	5.8	93.2	5.1	16.3	78.6
CB	3.9	34.2	61.8	12.5	30.4	57.1
CR	5.5	27.4	67.1	14.6	29.8	55.7
HS	2.9	28.2	68.8	5.8	27.4	66.8
HS sp	3.2	9.4	87.4	1.4	10.6	88.0

*We indicated extreme high and low values with bold and bold/italics, respectively. Values are mean and rounded values from the papers cited in the Results section. Variance is not shown.*

**Figure 1C** demonstrates that the dense accumulation of GABAergic terminals in and around str. pyramidale and a weaker, secondary increase in str. lacunosum-moleculare matches the perisomatically concentrated inhibition as well as the elevated inhibitory ratio in the distal apical dendrites.

### Hippocampal Pyramidal Cells Form Single Connections on Their Targets, While Inhibitory Cells Form Multiple Ones

To properly model and understand the effect of the convergent inputs, we summarize the termination strategy of excitatory and different types of INs over a single CA1PC. **Figure 1B** shows that hippocampal PCs contact their target neurons typically through a single synapse, regardless of the target’s type (Gulyás et al., 1993b; Sik et al., 1993; Arancio et al., 1994). In contrast, all examined types of IN formed multiple contacts on their targets cells (Gulyás et al., 1993a, 1996; Miles et al., 1996). Neurons terminating in the perisomatic region (on the soma and proximal dendrites), such as basket and axo-axonic cells, position 4–8 synapses close to each other onto the soma and proximal dendrites, and the axon initial segment of the innervated pyramidal (and inhibitory) cells, respectively. INs terminating on different dendritic domains, such as the bistratified or O-LM cells, also establish multiple synapses; however, these are scattered individually on different branches of the target neuron. Clustered inhibitory synapses on the dendrites of target cells can be found only in the case of interneuron-selective inhibitory cells such as the CR and/or VIP neurons (Acsady et al., 1996; Gulyás et al., 1996) and the HS cells (Gulyás et al., 2003). These cells establish climbing contacts on the dendrites of target INs.

### Properties of the Four Types of Neurons Selected for Modeling

The various types of hippocampal neuron are known to have different electrotonic structure (Emri et al., 2001). Synaptic conductances generate EPSPs with different amplitudes locally and voltage signals propagate and attenuate differently in distinct types of rat CA1 area INs. More recently, morphological and synapse distribution data became available from more interneuron types as well as from PCs (see above). Here, we will focus on how the morphology-dependent dendritic attenuation of synaptic signals and the distribution of excitatory and inhibitory synapses over different domains affect the accuracy and interpretation of VC measurements in these different neurons.

We selected four different types of CA1 neurons to be modeled in order to compare their signal propagation properties. A PC as well as a PV-, a CCK-, and a CR-positive interneuron were chosen from our 3D morphology database^1^ (also present at NeuroMorpho.Org under “Hippocampal cells from Gulyas”) to be modeled, using passive membrane conductances, synapse distribution, and activation frequency during different levels of activity, based on measurement of neuronal participation in different *in vitro* and *in vivo* observed activity states (see Materials and Methods). We chose these neurons because they are distinct in input organization. PCs are the principal neurons of the CA1 area, constitute 80% of neurons in this region and have a very characteristic dendritic arborization pattern. PV cells were included because they are important in controlling the action potential generation of PCs by providing perisomatic inhibition as basket and axo-axonic cells. They are similar to the PCs in respect of the large number of inputs (32000 for PCs, 16000 for PV) and in the low ratio of inhibitory inputs (5.3 versus 6%). The CCK and CR cells have significantly fewer inputs (8200 and 3800, respectively). CCK cells were selected because they receive the highest percentage of inhibitory inputs (36%) among all cells. CR cells were included because, although their input organization is similar to that of CCK cells, they have considerably thinner dendrites, which results in distinct electrotonic properties. We did not include the HS cell types because there is no available 3D dendritic morphology for them.

**FIGURE 3:**
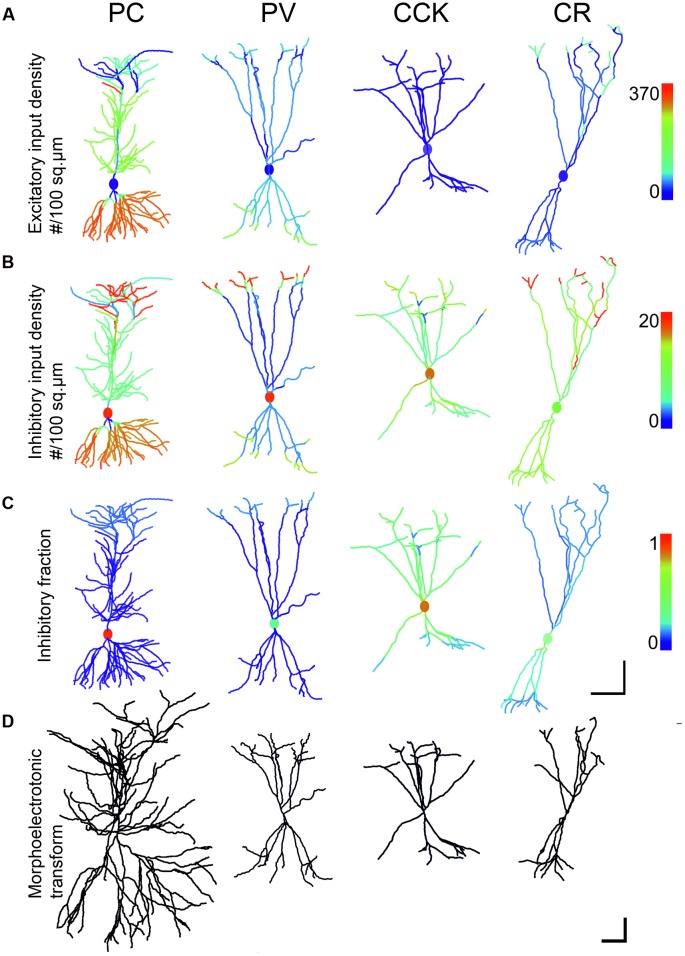
**Comparison of input density, excitatory to inhibitory ratio and morphoelectrotonic length of modeled neurons. (A,B)** The first and second rows show the excitatory and inhibitory input densities per membrane surface for the four examined cell types, presented on the same color scale for the same type of inputs. These values were used in the simulation when calculating convergent synaptic inputs in different states. Note that the PC receives the densest excitatory input onto its second order dendrites. **(C)** The third row shows the ratio of inhibitory inputs over the compartments. Inhibition is centered on the soma for all neurons. PC perisomatic regions get almost exclusively inhibition. CCK cells receive the highest amount of inhibitory input. **(D)** The fourth row shows the morphology of neurons after morpho-electrotonic transformation taking into consideration dendritic diameters. PCs are the least compact due to the small diameter of the second order oblique basal and apical dendrites that constitute the majority of dendritic compartments. Interneurons are more compact, due mainly to thicker higher-order dendrites. Scale: **(A–C)** 100 μm; **(D)** 1 lambda (electrotonic length constant).

The measured spatial distributions of excitatory and inhibitory inputs as well as their ratio over the surface of the four modeled neurons are shown in **Figures 3A–C**. The fourth row (**Figure 3D**) shows the morpho-electrotonic transform of the neurons’ geometry, where a unit length of dendrite corresponds to the distance over which the amplitude of a 100-Hz sinusoidal signal spreading toward the soma is decreased e-fold [this frequency, which is equivalent to a period of 10 ms, corresponds roughly to the characteristic time scale of fast synaptic currents, whose decay time constants are around 3–10 ms (Magee and Cook, 2000; Bartos et al., 2002)]. This method visualizes the electronic compactness of the neurons, calculated from dendritic length and thickness data in the resting state when only leak conductances are taken into account.

Pyramidal cells proved to be the least compact and CCK interneurons the most compact, despite their similar dendritic lengths. The origin of the difference is that PCs and INs bear dendrites with different arborization patterns. The overwhelming majority of the PC excitatory input arrives onto thin (0.25–0.55 μm) second order dendrites in str. oriens and radiatum, which are anchored to thick (1.0–2.5 μm) primary basal dendrites or to the substantial main apical dendrite (Megías et al., 2001). The main apical dendrite, due to its large diameter (2.5 μm), has a small axial resistance, and is therefore electrotonically continuous with the soma (it appears very short in the morphoelectrotonic transform of **Figure 3D**).

In all inhibitory cells the dendrites branch into daughter branches which have similar diameters. At the branch points the drop in diameter is smaller than in the case of the PC dendrites, with the result that while the primary IN dendrites are thinner (1.3–2.5 μm) than the main apical dendrite of the PC, the secondary and higher order dendrites have thicker diameter (0.5–2.0μm) than the second order ones in the PC (Gulyás et al., 1999; Mátyás et al., 2004). The end result is that PCs have electrotonically long dendrites that are not effectively coupled to the soma while the dendritic arbor of INs is considerably more compact electrotonically (**Figure 3D**).

Note that when synapses are activated at realistic rates the conductance of the membrane and thus the electrotonic compactness of the neurons change (see below).

### Estimation of the Tonic Membrane Conductance in Different Network States

Signal propagation in dendrites is heavily influenced by ongoing network activity because the activation of (either excitatory or inhibitory) synaptic inputs can substantially increase the average (tonic) membrane conductance, which in turn affects both the local response to synaptic input and the attenuation of (voltage and current) signals through the dendritic tree (Destexhe et al., 2003). In addition, the balance of excitatory, inhibitory, and intrinsic (voltage-gated and leak) currents also determines the (location-dependent) steady-state membrane potential, which influences the magnitude of synaptic currents through its effect on the driving force.

In order to examine this effect quantitatively, we estimated the (synaptic and leak) conductances per unit membrane area in three different network states for the four cell types we modeled (**Table 2**). As many *in vitro* physiological studies in single cells have been carried out under essentially silent network conditions, we also defined this case as our baseline condition (silent state, SS). On the other hand, our recent studies using an improved, spontaneously active hippocampal slice preparation (Hájos et al., 2009) allow us to estimate the firing rates of various hippocampal cell types during and between repetitively occurring SWR events. These high and low activity states will be referred to from here on, from the viewpoint of neurons, as high- and low-conductance states (HCS and LCS), respectively. We use the observed firing frequencies as the rates of activation of the corresponding synaptic inputs onto our model neurons. We then combine this information with the experimentally measured density of excitatory and inhibitory synapses, and the estimated maximal conductance and kinetics of single synapses, to calculate the mean excitatory and inhibitory synaptic conductance per unit membrane area for each part of the model neurons (see Materials and Methods, for more details). As we use a passive membrane model for our neurons, we assume the same basic leak conductance in all three states.

**Table 2:** Components of conductance at different activity levels for the examined neuron types.

	PC			PV			CR			CCK		
State	silent	LCS	HCS	silent	LCS	HCS	silent	LCS	HCS	silent	LCS	HCS
*g*_E_	0	67.4	269.5	0	33.7	134.8	0	5.9	23.6	0	12.5	50.1
*g*_I_	0	85	169.9	0	61.4	122.9	0	35.6	71.2	0	144.3	288.7
*g*_pas_	14.5	14.5	14.5	11.5	11.5	11.5	3.2	3.2	3.2	4.2	4.2	4.2
*g*_tot_	14.5	166.9	453.9	11.5	106.6	269.2	3.2	44.7	98.0	4.2	161.0	343.0

*The values are in nS.*

**FIGURE 4:**
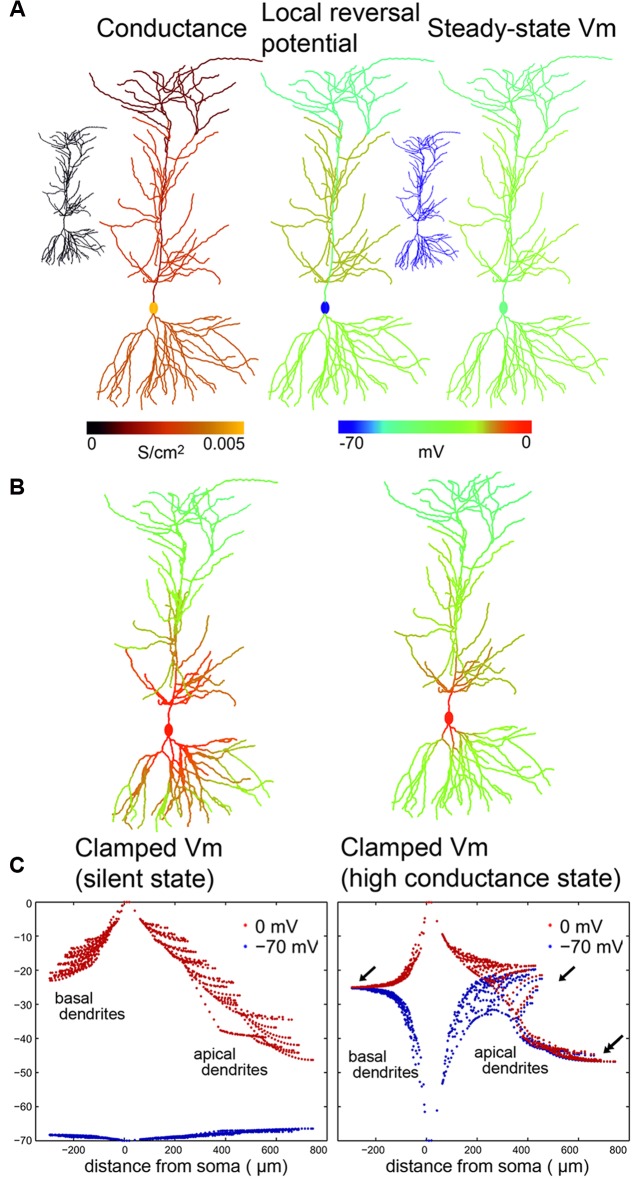
**Calculated voltage clamp (VC) errors in silent and high conductance states in a CA1 PC with passive membrane properties. (A)** Left: Conductance distribution (synaptic plus leak) over a simulated PC in high-conductance state (HCS), which results from activation of excitatory and inhibitory synapses with the measured distribution at a realistic rate. Center: Spatial distribution of the local reversal potential for the net membrane current. Right: The resulting location-dependent equilibrium potential from a simulation of the cell with local conductances and reversal potentials given by the previous two panels. The two small insets on the left and right show that, in the silent state (SS), all of these values are homogeneous over the cell (and both the local reversal potential and the equilibrium potential equal the global resting potential everywhere). **(B,C)** Membrane potential distribution over PC compartments in the presence of somatic VC in the SS (left) and in the HCS (right) are presented in two alternative ways. **(B)** is a color representation, while **(C)** shows the distribution of membrane potential as a function of distance from the soma. While **(B)** shows the potentials when the soma was clamped to 0 mV, **(C)** shows the distribution for two clamp potentials: –70 mV (blue) and to 0 mV (red). Note that the space clamp error quickly increases and has much higher absolute values when the soma is clamped to 0 mV, due to the larger difference between the local resting membrane potential and the clamping target potential. In the HCS the basal and apical dendrites are pulled toward approximately –25 mV (arrows) while the apical dendritic tuft is pulled toward –45 mV (double arrow) due to the higher ratio of inhibitory inputs in these distal dendrites.

Combining the effects of all conductances (with the appropriate reversal potentials) at the three different examined network states (SS, LCS, and HCS), we estimated the net local conductance density (by adding the contributions of synaptic and leak conductances) and the local reversal potential of the net membrane current (by taking the average of the reversal potentials of synaptic and leak currents, weighted by their respective conductances) over all compartments. This is illustrated for the PC in the left and middle columns of **Figure 4A**, respectively. The right column plots the steady-state voltage distribution in the cell, measured in a simulation after the end of initial transients, using the calculated values of the net conductance and the local reversal potential (the latter would be identical to the voltage distribution of the cell in the absence of axial currents). The estimated values of the synaptic, leak, and total conductances for each cell are listed in **Table 2**.

**Figure 4B** shows in color coding the membrane potential in different domains of a PC while the soma was clamped to 0 mV in the SS (left) or HCS (right). Further on we use another visualization (**Figure 4C**) that can more sensitively plot differences and allows plotting together several cases. The membrane potential values (*Y*-axis) at each model compartment are plotted against the distance of the compartment from the soma (*X*-axis). Red dots show how the membrane potential diverges from the clamp voltage away from the soma (located at zero) in the basal and apical dendritic tree of the PC when the soma is clamped to 0 mV. Blue dots show the steady-state voltage distribution when the cell is clamped to –70 mV.

Voltage converges toward the local equilibrium potential as we move away from the soma. At 0 mV holding potential, even in the SS (left), the clamp breaks down steeply. The breakdown in the SS is smaller when the soma is held at –70 mV, since it is close to the local reversal potential of the dendrites. In the HCS, the breakdown is very steep and affects both clamping potentials. This is because due to the simulated background activity the equilibrium potential of the dendrites derives from the excitatory and inhibitory reversal potentials weighted by the respective density of synaptic conductances and rates of presynaptic activation. At both clamping potentials, the distal dendrites converge to the local reversal potential set by the local *E*/*I* ratio. For the PC basal str. oriens dendrites and str. radiatum oblique dendrites converge to a more positive potential (arrows) than distal str. lacunosum-moleculare dendrites (double arrow), because on the latter ones the ratio of inhibition is higher. The efficacy of space clamp in the LCS state was qualitatively similar to that in the HCS state, although the equilibrium voltages were slightly different, and the breakdown was slightly less steep (data not shown).

### Factors Influencing the Breakdown of Voltage Clamp Efficiency

The factors which determine the synaptically evoked current as measured by somatic VC are as follows: (1) the conductance change evoked by the synaptic input; (2) the local driving force, which is the difference of the reversal potential for the appropriate type(s) of synaptically activated ion channels (which in turn is a function of the extra- and intra-cellular ion concentrations) and the local potential resulting from (partial) voltage clamping (these two factors determine the local amplitude of the synaptic current); (3) the attenuation of synaptic current from the site of the synapse to the soma.

The parameters of somatically measured synaptic events also depend on the spatial distribution of synapses (see above), and the distance-dependence of synaptic conductance as well as the rates of activation for these synapses. As a first step, we can convolve the synapse densities with location-dependent measures to get anatomically weighted averages or sums. As a second step, if we estimate the conductances for each type of input and their presynaptic firing rates in a given state (such as during and between SWRs), we can calculate anatomically and physiologically weighted averages of measured synaptic parameters for any given set of presynaptic firing rates, and thus for any given network state.

**FIGURE 5:**
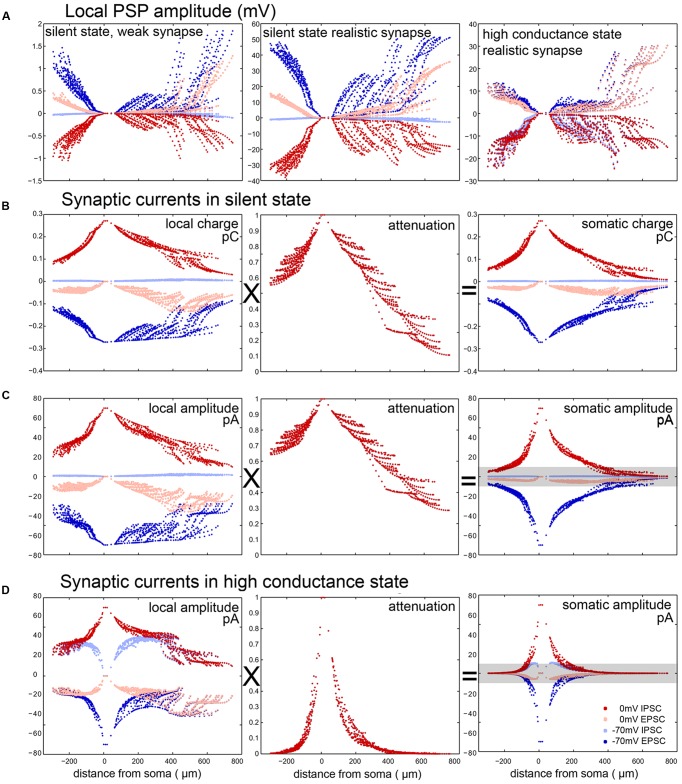
**Elements contributing to the error in the somatic measurement of postsynaptic current amplitude and charge in a PC. (A)** PSP amplitudes evoked in the SS by a single weak (left, maximal conductance: 10 pS) or a single realistic (center, 1 nS) synapse and by a realistic synapse in the HCS (right). The fact that the figure in the middle is not simply a scaled version of the figure on the left shows the sublinearity due to the effect that the local membrane potential approaches the synaptic reversal potential. In the HCS the input resistance drops, and therefore the voltage change is relatively smaller. **(B,C)** Somatically recorded PSC amplitudes and charges for single inputs (1 nS) in the SS are the result of the multiplication of local charge/amplitude values with the attenuation of charge/amplitude from the input site to the soma. **(D)** Somatically recorded PSC amplitudes are calculated similarly in the HCS. Note that here both local values and attenuation show a stronger drop away from the soma, due to high background conductance. Especially in the SS, EPSCs can be recorded with better accuracy (dark blue) and with smaller residual IPSCs (light blue) by clamping the cell to –70 mV, than IPSCs by clamping the cells to 0 mV (dark red), when there is a large amount of residual EPSCs (light red). This is due to the fact that the reversal potential of excitation is further away from the resting potential than the reversal of inhibition and thus the VC error is bigger when we try to measure IPSCs at 0 mV. Gray bar in **(C)** and **(D)** show those events that are below a specific PSC detection threshold (10 pA).

It is important to note that, especially for large synaptic conductances and thin distal dendrites, the locally evoked PSP can significantly reduce the driving force and thus decrease the current. Therefore, the amplitude of the local synaptic current, and the amplitude of the local PSP itself, do not scale linearly with the maximal synaptic conductance, but saturate for large conductances. This is clearly seen when comparing the left and central panels of **Figure 5A** – while the (realistic) conductance used in the middle panel is 100-fold larger than the small (probe) conductance in the left panel, the locally evoked PSP amplitude is not proportionally larger, and the ratio of the PSP amplitudes is smaller for more distal synapses which evoke larger responses.

Therefore, the main factors which affect the accuracy of the measurement of synaptic currents for somatic VC measurements are as follows. First, the efficiency of (static) VC breaks down in more distal dendrites, so the actual driving force is different from the nominal one. If the usual protocol is used (whereby the soma is clamped to –70 mV to suppress IPSCs and thus measure EPSCs, or to 0 mV to suppress EPSCs and thus measure IPSCs, assuming a reversal potential of –70 mV for inhibition and 0 mV for excitation) and the resting membrane potential is somewhere in between, then the actual local driving force is non-zero for the theoretically suppressed type of input, and smaller than the nominal value for the type of input we want to measure. As the resting membrane potential is usually closer to the inhibitory reversal potential than to the excitatory one (at least under silent conditions), the distortion is stronger when we clamp the soma to 0 mV. Thus, distal inhibitory currents are weaker than predicted by the single-compartment scheme, and are heavily contaminated by distal excitatory currents. Second, somatically measured PSCs are substantially attenuated compared to the local ones evoked in distal dendrites. As a result, somatically measured PSCs show a strong and non-trivial dependence on the location of the synapse (**Figures 5B,C**). Their amplitude is influenced by the interaction of distance dependent holding potential breakdown and the synaptic reversal potentials.

All of these effects depend heavily on the membrane conductance, which is in turn partly determined by the amount of other (“background”) synaptic input. To illustrate this point, we repeated the simulated VC experiments in the HCS. As expected, steady-state VC deteriorated further, and the attenuation of current/charge increased further compared to the SS (compare various measures between the SS and the HCS for PCs in **Figures 5C** vs. **5D**).

### The Breakdown of VC Efficiency and the Recovery of Excitatory versus Inhibitory Events Depend on the Cell Type and the Level of Neuronal Activity

**FIGURE 6:**
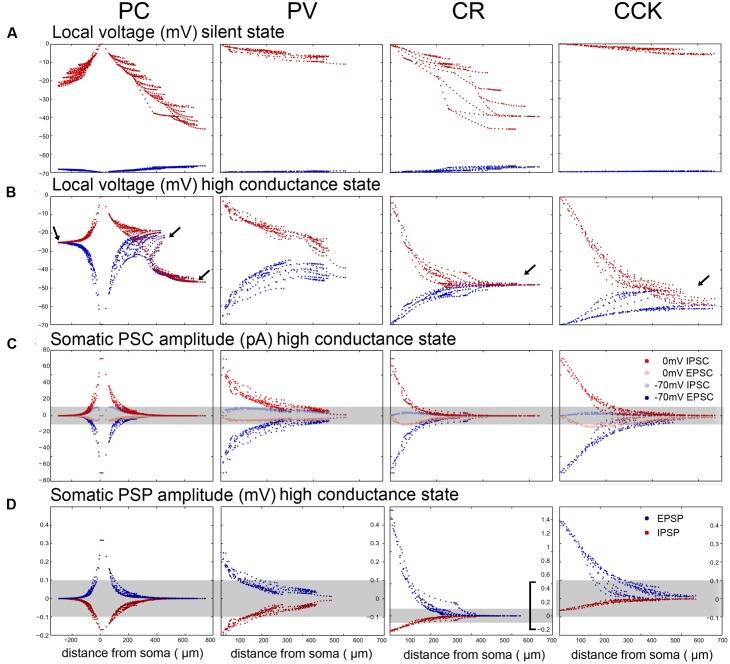
**VC measurement errors and somatic potentials are different in the 4 modeled neuron types. (A)** The somata of the model neurons were clamped either to –70 mV (dark red) or to 0 mV (dark blue) in the SS to show the breakdown of local clamped potential in different compartments. The breakdown is more severe when trying to clamp to 0 mV in all cell types, and it is much stronger in the electrotonically extensive pyramidal and CR cells than in the compact PV and CCK cells. **(B)** The breakdown of VC is considerably stronger in the HCS for all cell types. In the compact PV neuron the error is still relatively small. However, for the other cells the voltage converges over a short distance toward the equilibrium potential set by the local excitatory/inhibitory ratio (arrows). In the case of the relatively compact CCK cell the voltage converges toward a relatively negative potential because this cell receives the highest ratio of dendritic inhibition (see **Figure 3**). For PCs the convergence point is different for the oriens/radiatum dendrites and the lacunosum-moleculare dendrites, since the *E*/*I* ratio is different. **(C)** Comparison of somatically recovered EPSC and IPSC amplitudes at –70 and 0 mV (the results for charge are quite similar, and are therefore not demonstrated). As emphasized by the gray stripe covering PSC amplitudes below the 10 pA detection threshold, a significant portion of the synaptic inputs are not detectable due to the rapid loss of current as we move away from the soma. With the exception of PV cells, inputs further away than 100–150 μm are not detectable. **(D)** The distribution of somatically visible EPSP and IPSP amplitudes without somatic current injection (the somata sit at slightly depolarized membrane potentials where the interaction of excitatory and inhibitory bombardment clamps them). The gray stripe shows the PSP detectability threshold, which was set to 0.1 mV. Note that the scale is the same for the PC, PV, and CCK cells, but it is different for the CR cells that receive half a magnitude higher PSPs, due to the electrotonic organization of their dendrites.

Comparing the predicted efficacy of VC measurements in our simulations, we found that space clamp was best in PV neurons and worst in PCs and CR cells (**Figure 6A**). These differences are strongest in the HCS, where in all cell types except PV cells, distal dendrites essentially fully escaped somatic VC (**Figure 6B**). The dendritic equilibrium potential in active states (LCS and HCS) was strongly cell type-dependent as a result of differences in the ratio of excitatory and inhibitory inputs. Due to both space clamp errors and the increased attenuation of synaptic current, only proximal synaptic inputs remained detectable in all cell types (**Figure 6C**).

**FIGURE 7:**
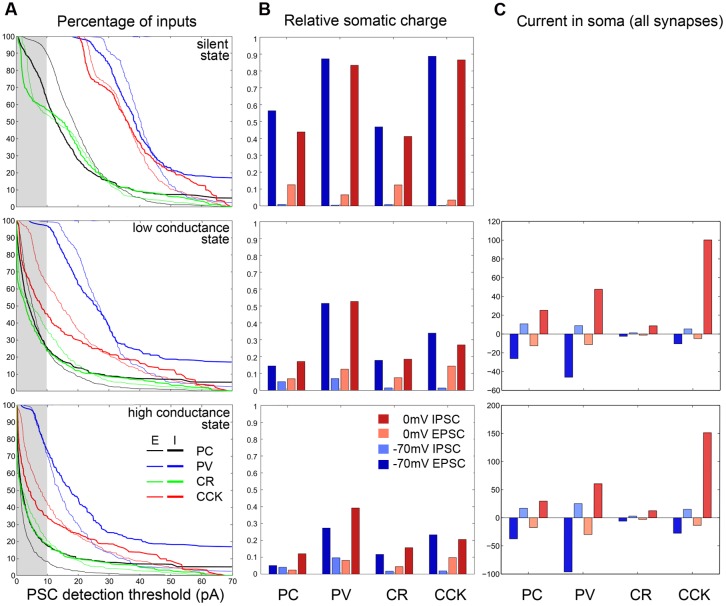
**Quantification of VC errors at different activity levels for the four examined cell types. (A)** The left column plots at three activity levels (first row: SS; second row: LCS; third row: HCS) for the four examined cell types (PC black, PV blue; CCK red; CR green) the cumulative amplitude curve for EPSCs (thin) and IPSCs (thick). The attenuation is strongest for PC and CR cells, weakest for PV cells. Note that as background activity and thus conductance increases, the curves shift to the left and more events disappear in the gray bar representing the 10 pA PSC detection threshold. **(B)** The average charge attenuation of excitatory and inhibitory inputs recorded at –70 and 0 mV, and weighted by synapse density (see Materials and Methods) are shown at different activity levels for the four cell types. In the non-realistic silent case excitatory and inhibitory events can be recovered reasonably well, though IPSC charge is contaminated by the relatively large amount of badly clamped EPSCs. In the more active cases, the attenuation is very strong for all cells (weakest for PV neurons). The same data are presented numerically in **Table 3**. **(C)** The right column shows the average excitatory and inhibitory current at the soma at –70 and 0 mV for the four cells, taking into account the synaptic densities and the estimated activation frequencies for these synapses in the active states. (The analogous plot is not shown for the SS where the average synaptic currents are zero due to the absence of presynaptic activity.)

Using these distance-dependent measures of the efficacy of somatic VC, and weighting these measures with the actual spatial distributions of synapses, we can now determine how different measures of ongoing synaptic activity are affected by VC errors. Synaptic events are usually detected using an amplitude threshold (whose optimal value depends on signal and noise amplitudes). Depending on the threshold, only a portion of synaptic events is detected. (Note that the PSC amplitude and thus detection also depends on the maximal synaptic conductance, which may also be distance-dependent, as observed in the main apical dendrite of CA1 PCs (Magee and Cook, 2000).) Plotting the cumulative distribution of PSC amplitudes in the SS, LCS, and HCS, we find that these differ very significantly across the different cell types (**Figure 7**). Assuming realistic synaptic conductances and detection thresholds, we find that very different portions of (excitatory and inhibitory) input are seen by somatic VC measurements. The portion of detected synaptic events is also severely dependent on the conductance state of the neuron – the majority of synaptic inputs are likely to be below the detection threshold in most cell types (other than PV cells) in the HCS. The highest proportion of synaptic events is visible in PV basket cells, and the lowest in PCs and CR interneurons – and the difference increases further in the HCS (**Figure 7**, left column; **Table 3**). Moreover, even for events above the detection threshold, the observed amplitude of the synaptic current is distorted in a way that depends on the cell type, network state, and distance from the site of measurement (see above).

**Table 3:** Percentage of PSCs detected with 10 pA threshold.

	EPSC			IPSC		
	silent	LCS	HCS	silent	LCS	HCS
PC	72.6	20.3	7.6	55.4	19.3	11.5
PV	100.0	98.0	52.5	100.0	96.6	63.1
CR	54.5	28.8	16.4	49.6	15.1	12.9
CCK	100.0	63.2	39.9	100.0	33.4	24.8

Rather than detecting and measuring individual synaptic events, synaptic activity is sometimes characterized by the net synaptic current, or by synaptic charge [the time integral of the measured synaptic current over a period of interest, (Oren et al., 2006; Hájos et al., 2013)]. This is also proportional to the average synaptic current over the same epoch. However, these measures are contaminated by synaptic currents of the opposite sign as explained earlier; due to imperfect space clamp, both excitatory and inhibitory synaptic events in distal locations contribute to the somatically measured current (and charge) at any holding potential. When the steady-state equilibrium membrane potential is close to the reversal potential of inhibitory currents (as is normally the case under silent conditions), inhibitory charge measured at 0 mV is especially strongly distorted by concurrent excitatory inputs. The asymmetry is likely to be weaker in the HCS where dendrites are more depolarized; in this case, due to the rapid breakdown of space clamp efficiency, synaptic charge measured at either 0 mV or –70 mV will contain substantial contributions from both excitatory and inhibitory synaptic input (see above). Comparing different cell types, we found that the largest proportion of (both excitatory and inhibitory) synaptic charge is visible in PV cells (followed by CCK cells), and the smallest proportion in PCs and CR interneurons (**Figure 7**, middle column).

As a next step, we estimated the amount of (excitatory and inhibitory) synaptic current that reaches the soma of a neuron in a given physiological (or pathological) activity state. This was done by weighting the distance-dependent measures calculated above by the maximal conductance and also by the rate of activation of each synapse, using our estimates of the presynaptic firing rates in the HCS and LCS as described earlier. It is important to note that there are a lot of unknowns in such an estimate of anatomically and physiologically weighted synaptic inputs; in particular, the maximal synaptic conductance values are rather variable and unknown for some sources of input, and the actual local values (not the ones which can be estimated by somatic VC, and which are often radically different from the correct local one, as shown above) are especially hard to estimate. However, using physiologically plausible ranges for the parameters (Bartos et al., 2002), there is a strong indication that the balance of synaptic excitation and inhibition is radically different in the cell types studied. In particular, using similar conductance values for the different cells and the firing rates measured during SWRs, we may estimate that while excitation and inhibition are approximately balanced in PCs and PV+ basket cells, CCK cells may be dominated by inhibition (**Figure 7**, right column). This is consistent with the data on SWR-related synaptic currents available for PV+ basket cells and CCK+ cells, and also with the observation that PV+ basket cells fire strongly during SWRs while CCK cells are less active or remain silent (Hájos et al., 2013); the lower excitatory-inhibitory ratio for PCs in the data is probably due to differences in the unitary synaptic conductance between cell types.

### The Propagation of Voltage Signals also Depends on Cell Type and Network State

**FIGURE 8:**
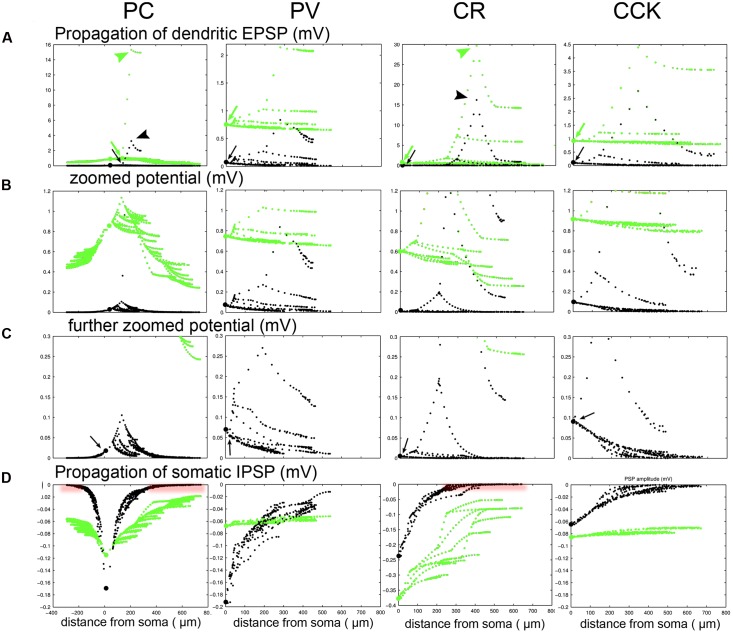
**Propagation of dendritic EPSPs demonstrates differences in integration complexity between PCs and different types of INs. (A)** A single excitatory synaptic conductance was placed on a mid-distal dendrite in each of the four cell types. The potentials in different domains of the cells were calculated and are shown for the SS (green) and the HCS (black). Somatic location is indicated by a larger dot. Note that the local potential is very large (green and black arrowheads) in the thin dendrites of the PC and the CR cells with a small drop toward the end of the local branch, and a steeper drop toward the soma. While the potentials hardly spread to other dendrites in the PC and CR cells they spread effectively for PV and CCK cells causing a steady depolarization throughout all compartments (green and black arrows). Both the local potential and the spread breaks down strongly in the HCS (black) compared to the SS (green). **(B,C)** The same as **(A)** but with enlarged scales for the potential to show what happens in the proximal/perisomatic region. A large drop is observed when the dendrite connects to the apical trunk (PC) or through a primary dendrite to the soma (CR), and then the potentials spread with only small further attenuation in the proximal dendrites/apical trunk. The attenuation of voltage signals is greatly enhanced in the HCS. In PV and CCK cells the local potential is smaller, but spreads effectively into all compartments of the neuron in the SS. The spread in the physiological HCS is still more effective for the PV and CCK cell (arrows in **C**), but now it fails for the distal compartments in these cells as well. **(D)** We also examined the effects of perisomatic inhibition. In the silent case inhibition completely propagates into all compartments for the PV and CCK cells and it spreads reasonably well in the PC and CR cell as well. However, in the HCS the spread is weaker; perisomatic inhibition still has some effect distally for PV and CCK cells, but totally vanishes beyond proximal compartments for the PC and CR cells (red shading).

We have now seen that the properties of synaptic inputs cannot be measured reliably using somatic VC recordings, especially in active, physiological network states. However, as the spiking output of the cell, as well as the activation of voltage-dependent mechanisms in the dendrites (which we have so far neglected), depend mainly on changes in membrane potential (rather than current), it is worth considering, at least briefly, how synaptically evoked voltage signals spread throughout the neuron, especially in the HCS. **Figure 6D** shows the magnitude of somatically measured EPSPs and IPSPs in the four cell types as a function of the location of the synapse, and confirms that a large portion of distal synaptic inputs may be undetectable in the HCS by somatic current clamp recordings as well. To examine this issue, we activated a single excitatory synapse in a mid-dendritic location in each cell, but now in a simulated “current-clamp” configuration (i.e., without clamping the somatic voltage). **Figures 8A–C** show that the largest EPSP can always be measured at the input site, and voltage always attenuates more strongly when it spreads toward the soma than when it spreads away from the soma, as predicted by cable theory (Koch, 2004). This is essentially because thin higher-order dendrites have much higher resistance (impedance) than thick proximal dendrites and the soma, with other dendritic branches from these compartments acting as further current sinks. However, there are also substantial differences between cell types. The local potential is large in the thin dendrites of the PC and the CR cells with a steep drop toward the soma. While the potentials hardly spread to other dendrites in the PC and CR cells they spread effectively for PV and CCK cells causing a steady depolarization throughout all compartments. Both effects are emphasized in the HCS compared to the SS. We also examined the spread of perisomatic inhibition. The fact that signal attenuation is much weaker in the outward direction means that perisomatic inputs (or any signal that reaches the soma) have a more global effect (Kubota et al., 2011, 2015), even in the HCS. In the silent case inhibition completely propagated into all compartments for the PV and CCK cells and it spread reasonably well in the PC and CR cell as well (**Figure 8D**). However, in the HCS the effect of perisomatic inhibition vanished beyond proximal compartments for the PC and CR cells. Thus, both excitation and inhibition have a more global effect on PV and CCK cells than on CRs and especially PCs. In the latter cells, excitatory inputs interact locally in distal dendrites, and, after spreading to the soma, are controlled by perisomatically acting inhibition.

### The Complex Interaction of Intra- and Extra-cellular Currents and Potentials

**FIGURE 9:**
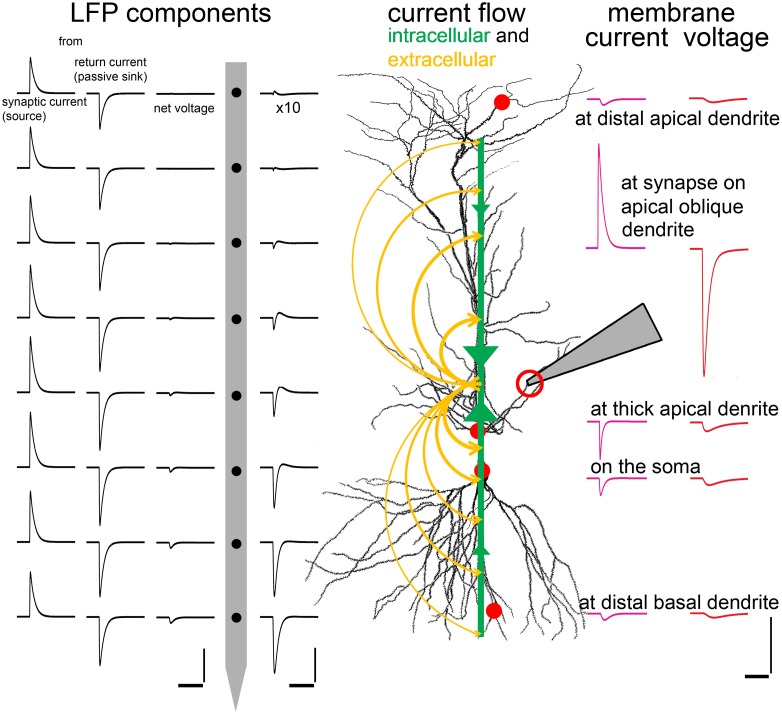
**Interactions among intracellular and extracellular signal components.** To demonstrate the intra- and extra-cellular spread of synaptic currents, a realistic inhibitory conductance (an active current source from an extracellular point of view) was simulated on a second order oblique dendrite of a multicompartmental CA1 model PC in str. radiatum, at the position indicated by the pipette and the red circle. Green arrows show the flow of intracellular current toward the synapse. Yellow arrows show the return extracellular currents (sinks) satisfying Kirchhoff’s current law. The current density drops away from the synapse. On the right side of the cell the intracellularly recordable potential (red) and membrane current (purple) are shown at different characteristic locations. Note that the current density (*I*_m_) is very large at the synapse due to its localized nature (red circle), and the local membrane potential change (*V*_m_) is also large due to the location of the synapse in a relatively high resistance, small, second order dendrite. Current and voltage transients attenuate away from the injection site. While the local potential change is negative at each location (red circles) the current reverses polarity as we move away from the site of the localized, large active current to the locations of the spread-out, passive counter-currents. On the left of the cell the extracellular signal elements are visible: the leftmost and the second column show the virtual voltage changes that would be evoked by active and passive currents, respectively, recorded with a virtual electrode array at regular intervals (100 μm). The third column shows the net LFP, which is equal to the summed voltage contributions of active and passive currents. Note that the large amplitude components very effectively cancel each other, so the layer-specific voltage differences can only be seen at 10 times magnification in the fourth column (between the electrode and the cell). The distribution of the voltage is asymmetrical because the majority of the return current is through the perisomatic area rather than the more distal apical dendrites. Therefore the “center of gravity” of the passive currents is shifted basally, forming a current dipole with the synaptic current, and producing an asymmetric voltage distribution. Scales: time scale is 20 ms for all scales, left: 5 × 10^–4^ μV, center: 5 × 10^–5^μV, right: 0.05 pA/μm^2^ and 0.5 mV, respectively, for the current and voltage trace.

Intracellular and extracellular currents and potentials interact in a complex and far from intuitive way, though the interaction is based on the simple laws of Kirchhoff. These complex interrelationships can be understood best by considering the intracellular and extracellular flow of currents. At the inhibitory synapse in **Figure 9** negative charge flows into the neuron, generating a localized outward current (as the direction of current flow is defined as the direction of movement for positive charge), which can be seen extracellularly as an (active) source at this point. The current then flows inside the cell in opposite directions along the neuronal processes. As it escapes the neuron through the membrane conductance of the processes, both the axial current and the membrane current decrease and the corresponding voltage drop across the membrane decreases, too. These distributed return currents are opposite in sign to the synaptic current (indeed, the sum of all membrane currents, including capacitive ones, over the entire surface of the neuron always equals zero due to the conservation of charge). Therefore, extracellularly, besides the active source, there will be passive sinks at the extremities of the neurons (these currents can be recovered from multielectrode voltage measurements using current source density analysis, CSD, (Buzsáki et al., 2012)). Since the active source is localized, the current density is high; in contrast, the opposing, passive currents are spread over a large surface and have small amplitude.

The intracellular voltage signal does not change polarity while its amplitude decreases as it spreads according to the rules of electrotonic propagation. In contrast, the total membrane current reverses sign as we move away from the input location and then its amplitude decreases similarly to the voltage signal. The extracellular potential shows a more complicated image. It results from the sum of contributions from all membrane currents (including both synaptic and return currents), where individual contributions are directly proportional to the amount of current and inversely proportional to the (3D Euclidean) distance between the current source and the extracellular measurement site. Therefore, the exact spatial profile of the extracellular potential depends on the 3D arrangement of neuronal processes, and the distribution of (active and passive) currents along these processes. Since the total membrane current for the entire cell is always zero, the field contributions of synaptic and return currents tend to cancel, especially at larger distances (see “LFP components” in **Figure 9**); i.e., the extracellular potential created by the neuron has no monopolar component. On the other hand, return currents are typically distributed asymmetrically in real neurons, giving rise to a substantial dipole component in the extracellular potential. In the example shown in **Figure 9**, where the synapse is located on a second-order oblique apical dendrite, a large portion of the return current flows through the soma rather than more distal portions of the apical dendritic tree, creating an effective current dipole.

### Interactions of Active and Passive Extracellular Currents in and among Modeled CA1 Pyramidal Cells

The 3D spread of the passive return currents is an essential element, because it interferes with the active currents in generating the LFP. If neurons were small spheres, the two currents would cancel out. If they were (finite) sticks, a relatively strong dipole would be formed. This is the representation that is often used in interpreting EEG results (Nunez and Srinivasan, 2005). However, PCs have a 3D tree-like structure, with apical and basal dendritic subtrees whose roots are at the soma, but which extend both horizontally (parallel with layer boundaries) and vertically (across layers).

**FIGURE 10:**
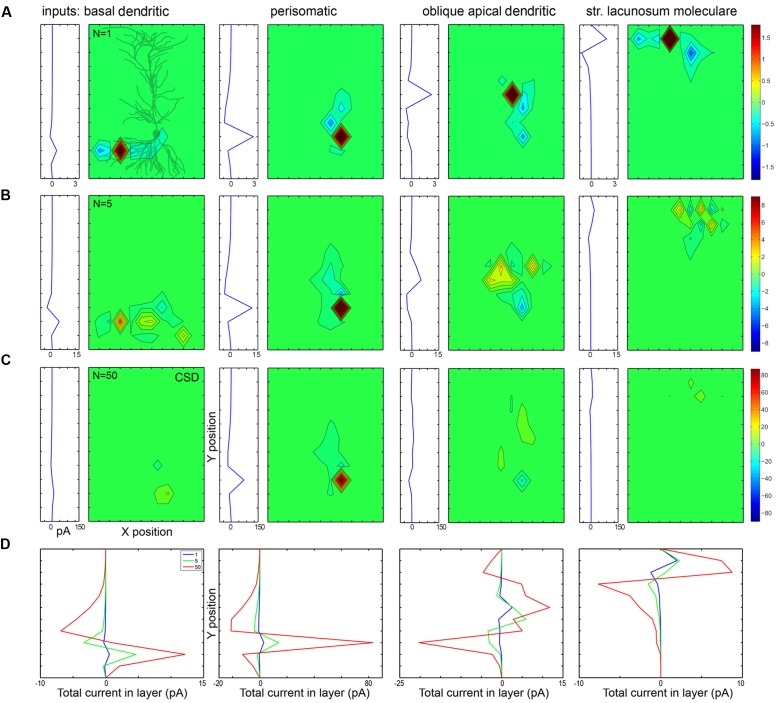
**Passive and active currents cancel when many synaptic inputs are distributed over the dendrites. (A)** When a single conductance is placed on different somato-dendritic compartments, the strong active current is surrounded by weaker passive currents spread out according to the orientation of dendrites. In each block the left graph shows currents recorded by a simulated electrode array from different layers, while the right (mostly green) panel shows the CSD in 2D (along the *X*- and *Y*-axes). In the first block a 2D projection of the modeled PC is shown in gray. **(B,C)** When synaptic inputs are scattered according to anatomical rules over the dendrites, the active currents of one input are canceled by the passive currents of the other inputs. In the case of 50 inputs **(C)** the cancelation is stronger than for five inputs **(B)**. For the centrally located, dense perisomatic inputs active and passive currents are consistently located within the same region and thus sum, resulting in a substantially stronger LFP signal than for the dendritic inputs. The scales are the same for all panels in a single row, and are changed in proportion to the number of inputs across rows. Numbers indicating color scales are in pA. **(D)** These panels compare the CSD depth profiles (shown individually in panels **A–C**) for different numbers of synaptic input (1, 5, or 50) to specific parts of the cell. Note the strongest summation for the perisomatic inhibition and canceling interactions for dendritic locations.

**FIGURE 11:**
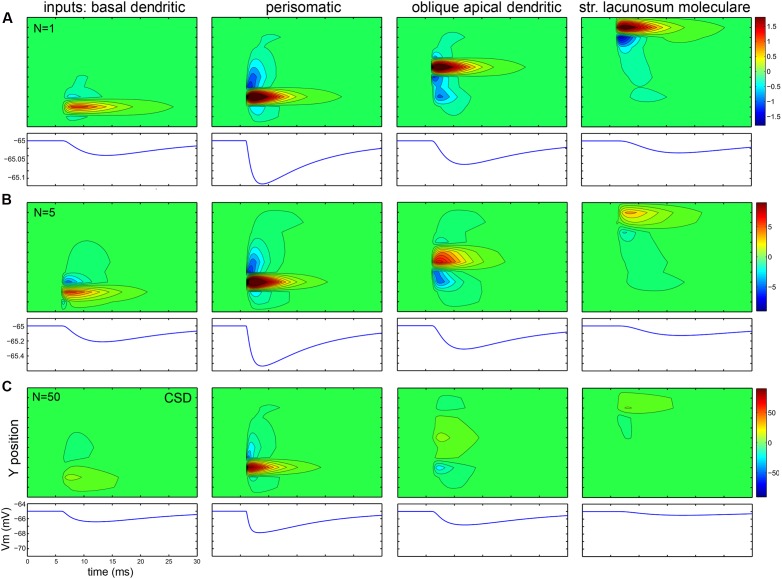
**While intracellular potentials sum, passive, and active extracellular currents cancel when many synaptic inputs are distributed over the dendrites. (A–C)** Data are presented similarly to the previous figure, but here the top panel shows the CSD across the layers versus time, while the lower panel shows the change of membrane potential versus time. The amplitude, rise and decay time of the events change according to the rules of intracellular signal propagation (amplitude decreases, rise and decay time increases for more distal events). Multiple inputs sum intracellularly, but for inputs other than perisomatic ones, the extracellular signals tend to cancel when the number of inputs is increased, resulting in a much weaker signal. Numbers indicating color scales are in pA.

The first row of panels in **Figure 10** plots in 2D (with *X*- and *Y*-axes parallel with and perpendicular to the apical dendrite, and collapsed along the *Z*-axis) the currents evoked by a single synaptic input located at different domains of a PC modeled in 3D. To the left of each 2D plot, the currents are summed within layers and plotted as a function of *X* location, representing what a CSD analysis of an electrode array (positioned parallel to the main axis of PCs) would show. **Figure 11** shows the effect of the same inputs, but here the currents in different layers are plotted against time. Below each plot the somatically recorded intracellular potential evoked by the inhibitory synaptic input is shown.

The first type of cancelation can be seen in the first and fourth panels of **Figure 10** in the case of a basal and a distal apical dendritic input (dendritic inhibition or excitation in str. oriens and lacunosum-moleculare, respectively). Here, the dendritic arbor has a significant horizontal spread, therefore the active current location (red) is flanked by passive currents (blue). When these are summed horizontally, the resulting amplitude of the current peaks is smaller than in the case of the somatic (second) or proximal dendritic (third) current, where the passive current extends mostly vertically.

The second, even more significant cancelation is shown in the second and third rows of the figure, where 5 and 50 synapses are distributed throughout the basal, somatic, proximal apical, and distal apical regions according to the observed distributions of axon terminals (**Figure 1B**).

Depending on the location of the synaptic inputs the interaction can be almost purely additive or strongly sublinear due to significant cancelation (**Figure 10D**). Somatically or perisomatically located inputs (basket and axo-axonic cell terminals) add almost linearly, resulting in an amplified response, while all other inputs (proximal and distal dendritic inhibition or excitation) interact in a strongly sublinear manner when 5, or, even more significantly, when 50 synapses are scattered (second and third rows), because these synapses are horizontally and vertically distributed and thus their active and passive currents cancel out.

Perisomatic synapses have an amplifying interaction for two reasons. First, multiple terminals of the basket cells are located close to each other at the electrotonic center of the PC from which dendrites fan out bidirectionally. Second, PC somata are aligned within a single layer (at least in the hippocampus). The active sources generated by perisomatic inhibition in str. pyramidale on the precisely aligned and abundant PC somata generate passive sinks in other layers; thus, both active and passive currents add up and thus strengthen each other as demonstrated in **Figure 10**. In the case of mid-dendritic synapses [such as Schaffer collaterals, bistratified INs, and neurogliaform/ivy cell terminals (Tamas et al., 2003; Fuentealba et al., 2008)] we can see strong cancelation, which is the result of two effects. First, when we superimpose currents from neighboring (horizontally shifted) cells (even if the synapses were placed at the same dendritic locations) there is a cancelation of dendritic currents within a layer horizontally, because active and passive currents from different cells overlap. The second type of cancelation arises from the fact that when synapses are located at radially different locations (whether on the same or different cells) they partially cancel each other vertically. As shown in the second and third rows of **Figure 10**, when we modeled multiple distributed synapses they canceled each other, resulting in a CSD signal which was one and a half magnitude smaller than the CSD for the focused perisomatic inhibition, although the intracellularly evoked voltage signal at the soma was comparable in magnitude.

A final component in the amplification of perisomatic and cancelation of dendritic currents derives from the fact that PCs do not receive perisomatic excitation, only very strong inhibition. Thus, there is no active current which could diminish the effect of inhibition perisomatically, but the scattered excitatory and inhibitory currents further interfere in the dendritic layers (unless they are temporally separated).

Comparing the different panels in **Figures 10** and **11**, we conclude that the intracellular summation of multiple inputs is equally strong for all input types, but extracellularly, basket cell terminals may generate an order of magnitude larger signal in str. pyramidale than a similar number of excitatory or inhibitory inputs in other layers.

## Discussion

### Mist in the Forest of Dendrites: We Do Not See Far

Experimental studies have shown that somatic and proximal dendritic intracellular recordings in pyramidal neurons miss a significant portion of synaptic inputs, save the perisomatic ones, especially at physiological (or pathologically high) levels of network activity (Williams and Mitchell, 2008; Marchionni and Maccaferri, 2009). We quantitatively extended these results for different cell types in the CA1 region of the hippocampus, and found that the effect is especially strong for PCs and CR-containing INs, where the majority of inputs are practically concealed. By contrast, PV+ INs have an electrotonically more compact dendritic tree and practically all of their inputs could be recovered with intrasomatic recordings.

In the case of extracellular potentials, we showed that as a result of the observed synapse distribution and complex interactions in 3D, perisomatic inhibition generates considerably stronger signals than excitation and dendritic inhibition. This is manifested in the fact that while field IPSPs generated by perisomatic inhibition have relatively large amplitudes (Bazelot et al., 2010), dendritic field IPSPs have smaller amplitude and are visible only after averaging 20–50 traces (Glickfeld et al., 2009). The detection of field EPSPs requires massive spike triggered averaging (Fernández-Ruiz et al., 2012).

The conclusions above may explain why perisomatic inhibition onto PCs had been deemed the most important factor in controlling PC activity. Our results also show that somatic whole-cell recordings and extracellular field potential recordings may lead to a biased view of the synaptic contributions to neuronal activity and network dynamics. More attention should be paid to how interactions of neurons in the network are affected by the dendritic computations implemented through the interaction of excitatory and inhibitory inputs, by integrating methods which can resolve these processes.

### Confounding Effects of Using a Passive Neuron Model and Identical Synaptic Conductances

A limitation of our study is that since detailed active conductance distributions are not available for all of the examined cell types, we used passive models whose parameters were based on commonly accepted values, or fitted using a few experimentally measured features such as somatic input resistance and time constant. The parameters and subthreshold behavior of our models were consistent with those in more detailed conductance-based models (Káli and Freund, 2005; Chiovini et al., 2014), and we believe that most of our conclusions are qualitatively and, in many details, quantitatively robust.

Incorporating active conductances in the models would have several effects. Any extra conductance will increase the space clamp and the attenuation error in VC, because it increases the apparent electrotonic length of the dendritic arbor. These effects will all augment the error of the current recovery. However, the level of augmentation can be cell type-specific, and thus the differences among cell types may shift when active conductances are taken into account.

The second effect is on the resting membrane potential, since many active conductances contribute to setting it. In the active case the potentials to which the dendrites settle in the presence of somatic VC (see arrows in **Figure 6B**) are probably different. Due to the activation of non-inactivating K^+^ conductances at more depolarized potentials the dendritic equilibrium potential might be more negative tonically, but the membrane potential could also be transiently more positive due to the activation of voltage-gated Na^+^ and Ca^2+^ channels and NMDA receptors.

The third effect is that some active conductances which are present in dendrites [e.g., Ih (Magee, 1998)] decrease the decay time of synaptic currents and thus make them appear faster. More rapid events propagate less effectively since dendritic processes work as a low-pass filter (Koch, 2004). Therefore, active currents may increase the loss of synaptic currents even further.

We also note that our reconstructed morphologies did not include dendritic spines, even for cell types (such as PCs) which are known to receive the majority of excitatory synaptic input onto dendritic spines. Adding spines to the models would increase the apparent membrane conductance, and would thus be expected to increase signal attenuation. On the other hand, moving synapses to dendritic spines would be unlikely to have a large impact on our results as spines have been shown to have an effect mainly on the amplitude of the local signal, and a much smaller effect on signals detected in other parts of the neuron, especially in models without voltage-dependent elements such as NMDA receptors and Ca channels (Gulledge et al., 2012).

In most simulations, we used a common value for the maximal synaptic conductance (1 nS), which is well within the physiological range. However, the actual value of the conductance is known to be pathway-specific, and is also quite variable even for a single pathway, partly due to the action of a variety of both associative and homeostatic plasticity mechanisms. We chose the same value for all synaptic conductances mainly because we wanted to focus on the effects of morphology and electrotonic structure, but also because the synaptic conductance has not been measured for many of the relevant pathways, and even reported values (mostly obtained using somatic VC) may easily be distorted by the effects described above. On the other hand, it is clear that both random and systematic variations of synaptic efficacy would have a substantial impact on our results. For instance, a systematic increase of the synaptic conductance with distance from the soma, as reported for the Schaffer collateral input to CA1 PCs (Magee and Cook, 2000), could compensate, at least within limits (Káli and Freund, 2005), for the increased attenuation of distal synaptic currents. Estimates of synaptic conductance based on anatomical measurements of synaptic dimensions (and the observed correlation between synaptic size and current) are becoming available for some cell types and inputs (Katz et al., 2009; Kubota et al., 2015), and can be taken into account in future studies. Furthermore, target-specific conductance values for projections would have an effect on the cell-type-specific balance of excitation and inhibition in active states. Random variability of the maximal conductance (i.e., variability not explained by cell type or synapse location) would have less effect on our results, indicated mainly by an increased variance in several measures.

### Why Are Centrally Located Inhibitory Inputs Lost at Nearly the Same Ratio as Distally Located Excitatory Ones?

Based on the somatically centered location of inhibitory inputs one would naively expect that IPSCs can be recovered more effectively than EPSCs. However, perhaps the most commonly used protocol to record IPSCs involves clamping the soma near the reversal potential of excitatory currents (0–10 mV), using an intrapipette solution which does not change the reversal potential of inhibitory currents. This clamping potential is far from the resting potential of the dendritic compartments and as VC breaks down (**Figure 4C**), the driving force is reduced and the amplitude of the IPSC is strongly attenuated. By contrast, to record excitatory inputs, the soma is usually clamped to the reversal of chloride (-70 to –55 mV), which is closer to the resting potential, and therefore the absolute clamping error due to imperfect space clamp is smaller in this case. However, in this case, the average attenuation is stronger, due to the more distal location of excitatory inputs. Thus, due to a different combination of effects, excitatory and inhibitory events can be recovered with comparable efficiency. Yet a small bias is present. As shown in **Figure 7B** the effectiveness of recovering excitatory and inhibitory currents depends on the background activity. Under realistic, high activity conditions, as observed in the papers studying the contribution of inputs to driving cells during HCSs (Hájos and Paulsen, 2009; Hájos et al., 2013), IPSCs onto PCs can be recorded 2 times more effectively than EPSCs. For PV cells this ratio is close to one. Finally, it should be noted that IPSCs can also be recorded at hyperpolarized clamping potentials using a high chloride pipette (usually while excitatory inputs are blocked pharmacologically). In this case, the error associated with imperfectly clamped dendrites should be substantially reduced.

A practical technical point that arises from our results is that, if one wants to sample the level and pattern of excitatory and inhibitory activity in a network, these events can be conveniently recorded from a PV neuron, because both types of input to almost any location in the dendritic tree are visible with relatively little distortion to a somatic VC electrode.

### The Functional Importance of Perisomatic Inhibition

Several recent studies concluded that perisomatic inhibition was important in the generation of different types of network activity, such as gamma oscillation and SWRs (Oren et al., 2006, 2010; Adesnik and Scanziani, 2010; Tahvildari et al., 2012; Hájos et al., 2013). In contrast, we argue here that the contribution of perisomatic inhibition to the measured synaptic current may be overemphasized by both intracellular (VC) and extracellular (field potential) recordings. In the intracellular case this is due to the dramatic decrease in the detectability of IPSCs away from the soma (see also, Kubota et al., 2015). In the extracellular case, while somatic input sums effectively, dendritic cancelation radically reduces the extracellular visibility of dendritic inhibition.

So the question arises: Is perisomatic inhibition really as effective and important as it seems? To answer this question it is important to distinguish its importance in generating prominent currents in intrasomatic or extracellular recordings, and its role in controlling neuronal activity (silencing, phase locking, and synchronization).

Current source density analysis and voltage sensitive dye recordings (Ylinen et al., 1995; Csicsvari et al., 2003; Mann et al., 2005) demonstrated that strong perisomatic currents and voltage changes are associated with both gamma oscillations and SWRs. A correlation has also been demonstrated between inhibitory currents and the extracellular field from str. pyramidale (Oren et al., 2006). These observations, however, did not prove causality.

More recently, several types of active manipulations of the system, such as localized or cell type-selective drug application (Foldy et al., 2007; Glickfeld et al., 2008; Gulyás et al., 2010; Schlingloff et al., 2014), cell type- or location-selective optogenetic silencing or activation (Ellender et al., 2010; Schlingloff et al., 2014; Stark et al., 2014), and local lesions impacting the axon of specific types of INs (Schlingloff et al., 2014), have been performed. Such active manipulations proved that perisomatic inhibition not only shapes the LFP (and therefore acts as a current generator), but it is an active agent in timing PC firing and shaping network activity (and is therefore an essential part of the rhythm generators) during both gamma oscillation and SWRs. The papers also identified PV-positive basket cells (and excluded AACs and CCK cells) as the cell type responsible for this action (Gulyás et al., 2010; Schlingloff et al., 2014). The fact that the collapse of PV+ cell-mediated inhibition results in uncontrolled firing during epilepsy (Karlócai et al., 2014) also supports their functional importance.

### Local Methods Are Required to Analyze the Importance of Dendritic Processing

The papers cited above implicitly suggest the rather boring image where neuronal output is controlled during all observed network patterns by PV+ neurons, especially basket cells. So what is left for other types of INs? Freund (2003) and Freund and Katona (2007) proposed that PV+ perisomatic cells are responsible for the rhythm, while CCK cells for the “mood,” i.e., PV+ cells control neuronal firing, phase and synchrony, while the CCK cells (many of them dendritic inhibitory) mediate subtle subcortical modulatory effects. The more recent papers described above support that PV+ cells serve primarily as clock cycle generators during different brain states. The robust inhibition of the PV+ basket cells can be easily detected by our somatically centered, “crude” methods. To reveal the more subtle function of non-PV+ neurons, which may, for instance, participate in modulation of dendritic integration and plastic processes by influencing Ca^2+^ spikes (Miles et al., 1996), we have to use more sensitive methods which can detect activity changes during behavioral state-associated transitions and interactions happening in the dendritic tree.

A series of elegant imaging studies has revealed PC dendritic summation, integration, and plasticity mechanisms (Losonczy and Magee, 2006; Losonczy et al., 2008; Makara et al., 2009; Lovett-Barron et al., 2012; Takahashi et al., 2012; Makara and Magee, 2013). Strong supralinear, localized interactions that engage active conductances and dendritic spike generation were revealed. However, most of these studies were limited to the analysis of excitatory input interactions only. A recent set of studies started to reveal the role of inhibition in the control of dendritic supralinear processes, using localized pharmacology and the combination of optogenetic stimulation and recording methods (Müller et al., 2012; Pouille et al., 2013; Müller and Remy, 2014). To reveal the subtle role of non-PV+ neurons these interactions will have to be studied during different brains states and state transitions.

### But Neurons Work in Current Clamp, Not in Voltage Clamp

Based on our results we can estimate the error of excitatory and inhibitory event recovery in VC in different states (**Figure 7B**; **Table 3**). In theory, we can calculate the total current arriving onto the neuron by multiplying the recovered currents (**Figure 7C**) with the relative errors (**Figure 7B**). We can also calculate the more relevant conductances (**Table 3**). But does it help us understand neuronal computations if neurons actually work in current clamp (i.e., to a first approximation, action potential output and dendritic active currents are generated when the membrane potential exceeds some threshold)? VC enables us to characterize excitation and inhibition separately, but a neuron sitting around rest and integrating potentials does not see them this way. A useful contribution of VC is that we can estimate the magnitude of concurrent conductances (using appropriate corrections), and thus the electrotonic compactness of a neuron.

The real question is to what extent different compartments of the neuron are visible to each other in current clamp? And, most importantly: What do the soma and axon initial segment see?

**Figure 8** demonstrates the extent of visibility in different compartments for distinct cell types. In the passive model, inputs onto an oblique PC dendrite evoke a large potential change which is restricted to that particular dendrite, although the signal can be substantially attenuated as it spreads toward the soma. When the signal enters the soma or the main apical dendrite, it is strongly attenuated (due to the low impedance of the thick compartment), but then it spreads without much further reduction into the whole complex compartment. This means that the electrotonically compact soma/apical dendritic compartment sums equally the signals from all of the converging oblique dendrites. The situation is different for the electrotonically more compact PV and CCK cells, where inputs effectively influence the potential of all soma-dendritic compartments, and thus potentially interact throughout the whole extent of the neuron. Input integration in CR cells is somewhere in between, as individual primary branches seem to be integration units that converge onto the soma.

### Outlook to Other Brain Areas

Finally, the question arises to what extent our conclusions can be extrapolated to neocortex or other brain areas.

Strong EEG signals are generated in laminated and ordered structures, such as the cortex or retina (Covey and Carter, 2015). Since cellular fields do not add up in a homogeneous structure such as the thalamus or striatum, these structures do not generate noticeable contributions to the electric field. Considering that in the neocortex principal neuron somata are organized into five layers (2–6) and principal cells of different layers have somewhat distinct morphology, we expect that the signals generate in the neocortex will be smaller than in the simpler and more organized hippocampus. Dendritic cancelation will be similar or stronger, due to an even more heterogeneous mixing of processes. The summation of perisomatic currents is expected to be weaker since the somata are not organized so regularly. Additionally, while in the hippocampus >53% of the basket cell terminals are located on the somata of PCs (Halasy et al., 1996), this ratio is only 33% in the case of neocortical basket cells (Somogyi et al., 1983). This further weakens the perisomatic concentration and thus summation.

On the other hand, signal propagation in other extended neuronal cell types is bound to be similarly influenced only by the length and diameter of the dendrites and the synapse distribution, and not by the actual 3D arborization. Therefore, the conclusions on intracellular signal integration should be similar, regardless of whether or not the cells are located in laminar and organized structures.

### Input Integration Is More Complex in PCs than in INs

The traditional one-step integrate-and-fire model is too simple to approximate the I/O function of all types of neuron. A decade of evidence indicates that a more complicated, multilayered, non-linear model is more appropriate to describe PC integration (Poirazi et al., 2003a,b; Polsky et al., 2004; Losonczy and Magee, 2006; Behabadi and Mel, 2014; Jadi et al., 2014). One of the conclusions of dendritic imaging papers (Losonczy and Magee, 2006; Losonczy et al., 2008; Makara et al., 2009; Lovett-Barron et al., 2012; Takahashi et al., 2012; Makara and Magee, 2013) was that in the dendritic tree of a PC a coordinated, non-linear interaction of spatially and temporally localized synaptic inputs evokes local, active dendritic currents (NMDA spikes, Ca^2+^ spikes, dendritic Na^+^ APs) that effectively spread to the soma/apical dendritic compartment. The possible importance of input clustering was highlighted by results which demonstrated that functionally related synaptic inputs are spatially arranged into clusters over second order PC dendrites, facilitating the interaction of relevant inputs (Kleindienst et al., 2011; Makino and Malinow, 2011; Takahashi et al., 2012; Glickfeld et al., 2013). Additionally, Cohen and Miles (2000) quantified an observation shared by many experimenters, namely, that only a small portion (22%) of PC action potentials are preceded by EPSPs, while this ratio is 95% for INs.

The above observations support a more complex, two-level PC input integration model. Individual excitatory events supralinearly sum in individual second order dendrites (first sum and threshold) and spread to the soma/apical dendritic compartment that sums the result of parallel computations of individual second order dendrites and initiates an action potential (second sum and threshold). Thus, action potentials in PCs are often not preceded by a somatically visible EPSPs, because most APs are initiated as a result of the summation of active dendritic currents.

The two-level integration model is also supported by the anatomical facts of PC architecture. First, the thick apical and proximal basal dendrites seem to be a functional extension of the soma (**Figure 8**, PC), conducting the output of the thin, second order oblique dendrites to the site of action potential generation. Second, while second order dendrites receive only a small proportion of inhibition, the somata and the proximal dendrites of PCs receive exclusively inhibitory input, and the amount of this inhibition is quite substantial.

The existence of perisomatic versus dendritic INs also supports the two-level integration model. It was suggested that the two types of inhibition control different aspects of input integration (Miles et al., 1996; Pouille et al., 2013).

Marchionni and Maccaferri (2009) showed that local, perisomatic inhibition only disconnects distal dendrites but does not shunt events in the dendrites. Similarly, Lovett-Barron et al. (2012) concluded that dendritic inhibition was more effective than perisomatic inhibition in regulating excitatory synaptic integration. This matches our finding, shown in **Figure 8D**, that perisomatic inhibition cannot effectively influence dendritic processing in PCs. Thus, dendritic INs control dendritic summation and non-linearities and are in control of the first integration level. By contrast, basket and axo-axonic cells, in a different process influence AP generation and timing and control the second level of integration.

Input integration in the case of PV and CCK INs can be described with a simpler, one-layer model. In these cells inputs spread effectively to the soma for several reasons: (1) they arrive onto dendritic shafts and not spines; (2) second-order dendrites are not much thinner than the first-order ones, and thus conduct signals more effectively than second-order PC dendrites; and (3) excitatory inputs arrive at the perisomatic domain, too.

Therefore, in PCs the local interactions of excitatory and inhibitory inputs within individual dendritic branches contribute fundamentally to the action potential output of the cell, while in the case of most INs even individual or spatially scattered EPSPs can drive the cell to spike. However, a complete understanding of the integrative properties of these different cell types will require a proper combination of anatomical, physiological, imaging, and computational approaches which can resolve the contributions of excitatory and inhibitory synaptic inputs as well as intrinsic non-linear processes to information processing within dendrites and the functional interactions between different cellular compartments in various physiological brain states.

## Author Contributions

AG: Concept and writing the manuscript; TF: Concept and writing the manuscript; SK: Concept, modeling, analysis, and writing the manuscript.

## Conflict of Interest Statement

The authors declare that the research was conducted in the absence of any commercial or financial relationships that could be construed as a potential conflict of interest.

## Footnotes

1 http://www.koki.hu/~gulyas/ca1cells/
